# Glyburide Suppresses Inflammation-Related Colorectal Tumorigenesis Through Inhibition of NLRP3 Inflammasome

**DOI:** 10.3390/ijms252111640

**Published:** 2024-10-30

**Authors:** Toshihide Maeda, Yohei Shirakami, Daisuke Taguchi, Takao Miwa, Masaya Kubota, Hiroyasu Sakai, Takashi Ibuka, Kosuke Mori, Hiroyuki Tomita, Masahito Shimizu

**Affiliations:** 1 Department of Gastroenterology, Graduate School of Medicine, Gifu University, Gifu 501-1194, Japan; 2 Department of Tumor Pathology, Graduate School of Medicine, Gifu University, Gifu 501-1194, Japan

**Keywords:** colorectal cancer, NLRP3, inflammatory bowel disease, glyburide

## Abstract

Colorectal cancer represents one of the most serious complications of inflammatory bowel disease. The NLRP3 inflammasome plays a pivotal role in the onset and progression of inflammatory bowel disease and is also implicated in colorectal cancer. This study aimed to investigate whether NLRP3 deficiency or glyburide, a sulfonylurea used for diabetes management and known as an NLRP3 inhibitor, could suppress colitis and its related colorectal tumorigenesis. Mice were divided into three groups: a control group, a glyburide group, and an NLRP3-deficient group. We investigated acute colitis and inflammation-related tumor models using azoxymethane and dextran sodium sulfate. In the colitis model, the colonic inflammation grade was significantly increased in NLRP3-deficient mice but not in mice administered glyburide. In the colorectal carcinogenesis model, fewer colorectal tumors were observed in both NLRP3-deficient and glyburide-treated groups. Additionally, a reduction in the expression levels of inflammatory cytokine genes was detected in the colonic mucosa of the mice of these groups. These findings suggest that NLRP3 deficiency may exacerbate acute colitis, while pharmacological inhibition, as well as deficiency of NLRP3, suppresses colitis-related tumorigenesis, presumably due to the attenuation of chronic inflammation in the colorectum. Glyburide holds promise as a potential chemopreventive agent for colitis-related colorectal cancer.

## 1. Introduction

Colorectal cancer (CRC) is the third most diagnosed cancer and the second leading cause of cancer-related deaths worldwide [1]. Despite advances in technology for early detection and treatment, CRC remains a significant health concern. Additionally, CRC presents as one of the most serious complications of inflammatory bowel disease (IBD), including ulcerative colitis and Crohn’s disease, both of which have an increasing incidence; therefore, the number of patients with IBD-related CRC is expected to rise [2].

Inflammasomes are intracellular molecular complexes primarily expressed by innate immune cells that control the initiation of inflammation in response to danger signals associated with infection or injury, playing crucial roles in innate immunity. The Nucleotide-binding oligomerization domain, Leucine-rich Repeat and Pyrin domain-containing 3 (NLRP3), consists of NLRs, caspase-containing apoptosis-associated speck-like protein (ASC), and procaspase-1. Upon activation of the NLRP3 inflammasome, NLRP3 proteins recruit the adapter ASC and procaspase-1 for cleavage and activation, leading to the maturation and secretion of inflammatory cytokines and proptosis [3]. Activation of the NLRP3 inflammasome is implicated in the onset and progression of inflammatory diseases, with elevated NLRP3 expression reported in patients with IBD [4]. Furthermore, the expression of NLRP3 in CRC tissue serves as a poor prognostic factor [5,6], suggesting that the regulation of NLRP3 may hold therapeutic value for IBD and its related CRC.

Sulfonylurea glyburide (GLB), also known as glibenclamide, is an oral medication used for individuals with type 2 diabetes. This medication is commonly prescribed in clinical practice, but its side effects, including hypoglycemia, weight gain, nausea, heartburn, bloating, and hemolysis associated with glucose-6-phosphate dehydrogenase deficiency, should be noted [7]. Inhibition of ATP-dependent potassium channels by GLB in pancreatic β-cells leads to enhanced insulin secretion. GLB is also reported to operate upstream of cryopyrin and downstream of P2X7 receptors, resulting in the suppression of NLRP3 inflammasome activation [8].

This study aimed to elucidate the potential preventive effects of GLB on colitis-related colorectal tumorigenesis. To achieve this objective, murine models of dextran sodium sulfate (DSS)-induced colitis and azoxymethane (AOM) plus DSS-induced CRC were employed, which closely resemble human inflammation-related colitis and colon carcinogenesis, respectively. The investigation focused on assessing whether the oral administration of GLB inhibits colitis and its associated tumor development in the inflamed colorectum by modulating NLRP3, with NLRP3-deficient mice serving as the control group.

## 2. Results

### 2.1. Experiment 1 (DSS-Induced Colitis Model)

#### 2.1.1. General Observations

**Figure 1:**
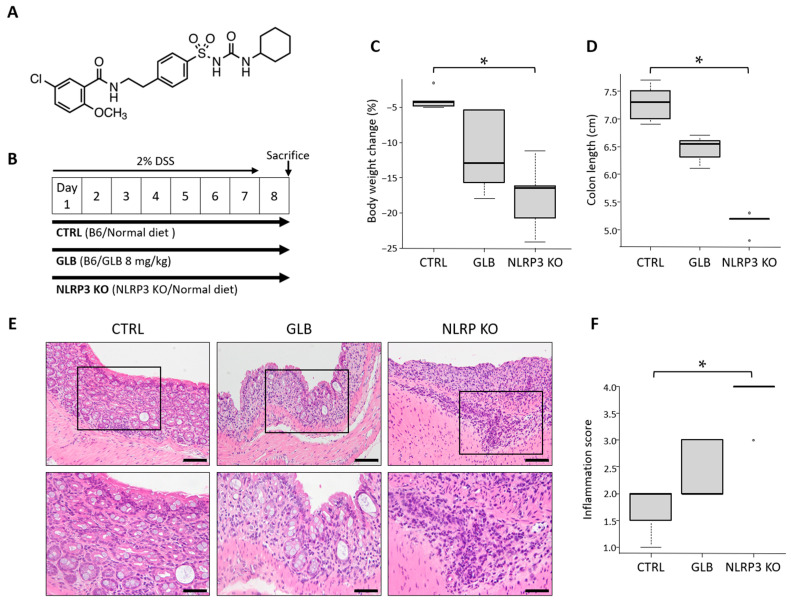
Effects of glyburide or NLRP3 deficiency on colitis induced by DSS (experiment 1). (**A**) Chemical structure of glyburide. (**B**) Study protocol of DSS-induced colitis. (**C**) Body weight change in the experimental mice. Percentage of weight loss at the end of the study compared to the beginning. (**D**) Colon length measured from the ileocecal junction to the anal verge. (**E**) Representative photographs of colon sections stained with hematoxylin and eosin. Enlarged pictures of the section enclosed within the square in the upper panels are shown in the corresponding lower panels. Scale bars: 100 µm for upper panels and 50 µm for lower panels. (**F**) Inflammation scores. Data represent mean ± standard error. * *p*  <  0.05. CTRL, control (C57BL/6J) mice; DSS, dextran sodium sulfate; GLB, glyburide; KO, knockout.

The chemical structure of GLB is shown in Figure 1A. In the DSS-induced colitis model, mice were divided into three groups: control C57BL/6J (CTRL), NLRP3-knockout (KO) mice fed a control diet, and GLB-administered C57BL/6J mice; to all three groups, 2% DSS was administered in drinking water for 7 days (Figure 1B). In this experiment, body weight loss was noted in all groups and significant decreases in body weight and colon length were observed in the NLRP3-KO mice compared to those in the CTRL group (Figure 1C), which presumably reflected colorectal inflammation [4]. Although tendencies of decreased body weight and colon length were observed in the GLB-administered mice compared to those in the CTRL group, these differences were not statistically significant (Figure 1C,D). There was no significant difference in the relative weights of the liver and kidneys among the three groups. A histopathological investigation also revealed no alterations to these organs, suggesting no toxicity of NLRP3 deficiency or inhibition in this experimental model.

#### 2.1.2. Effects of GLB and NLRP3 Deficiency in DSS-Induced Colitis

The colonic inflammation grades at the end of the experiment were evaluated based on inflammation scores (Figure 1E,F). In NLRP3-KO mice, the inflammation scores were significantly higher than those in the CTRL group. However, administration of GLB exhibited no significant effect on the colonic inflammation grade when compared to CTRL mice. These findings suggest that DSS-induced colitis was exacerbated due to NLRP3 deficiency and that pharmacological inhibition of NLRP3 by GLB had no significant effect on acute colitis.

#### 2.1.3. Expression Levels of Inflammatory Cytokine mRNAs in Colonic Mucosa

**Figure 2:**
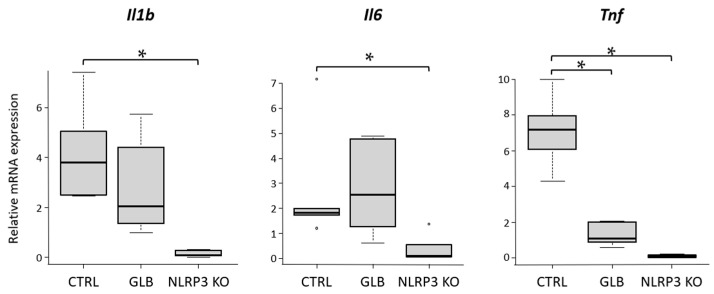
Effect of GLB and NLRP3 deficiency on expression levels of inflammatory cytokines in the colon of a mouse colitis model. mRNA expression levels of *Il1b*, *Il6*, and *Tnf* in the colonic mucosa were measured by qRT-PCR with specific primers. Data represent mean ± standard error. * *p*  <  0.05. CTRL, control C57BL/6J mice; DSS, dextran sodium sulfate; GLB, glyburide; KO, knockout.

The inflammatory cytokines, namely *Il1b*, *Il6*, and *Tnf*, in the colonic mucosa were assessed (Figure 2). Gene expression levels of these cytokines were notably diminished in NLRP3-KO mice in comparison to the CTRL group. In mice subjected to GLB treatment, *Tnf* levels were substantially reduced in the colonic mucosa, while no significant differences were observed concerning the other investigated cytokines when compared to the CTRL group.

### 2.2. Experiment 2 (AOM/DSS-Induced CRC Model)

#### 2.2.1. General Observations

**Figure 3:**
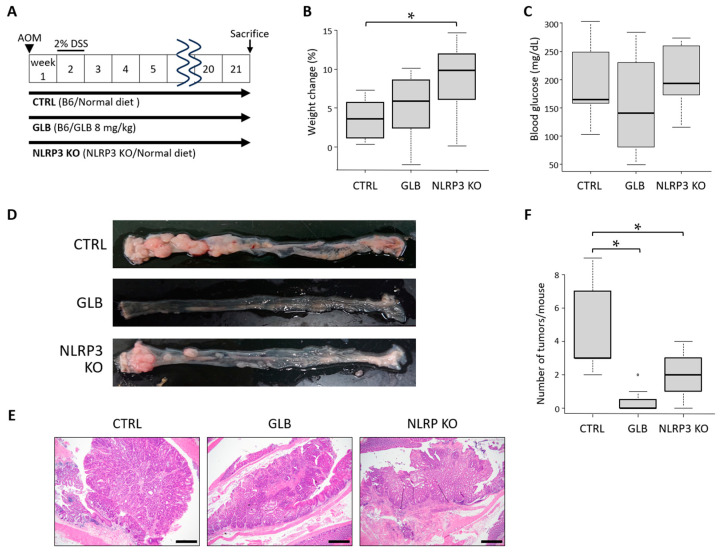
Effects of glyburide or NLRP3 deficiency on inflammation-related colon tumorigenesis induced by AOM/DSS (experiment 2). (**A**) Study protocol of a mouse AOM/DSS-induced CRC model. (**B**) Body weight change in the experimental mice. Percentage of weight gain at the end of the study compared to the beginning. (**C**) Blood glucose levels at the end of the experiment. (**D**) Representative macroscopic pictures of tumors in the colorectum of the experimental mice. (**E**) Representative pathological image of the colon tumor in each experimental group. Scale bars: 500 µm. (**F**) Number of tumors per mouse colon. Data represent mean ± standard error. * *p*  <  0.05. AOM, azoxymethane; CTRL, control C57BL/6J mice; DSS, dextran sodium sulfate; GLB, glyburide; KO, knockout.

To further explore the effects of NLRP3 deficiency or inhibition on colon diseases, we employed a mouse AOM/DSS-induced colorectal tumorigenesis model (Figure 3A). At the end of the experiment, while the mice in all groups had gained weight, those in the NLRP3-KO group showed marked weight gain compared to those in the CTRL group (Figure 3B). Similarly to the above DSS-induced colitis model, there were no significant alterations in weights and histological findings in the liver and kidneys among the three groups. Because GLB has an insulin secretion-stimulating effect, we checked the blood glucose level, but no significant difference was observed (Figure 3C).

#### 2.2.2. GLB Administration and NLRP3 Deficiency Suppress Inflammation-Related Colorectal Tumor Development

Macroscopic colon images revealed the development of tumors in the colorectum of the experimental mice (Figure 3D). Pathological examinations found that the colorectal tumors were classified as dysplasia, adenoma, or cancer (Figure 3E). The number of tumors in the colon was significantly reduced in the GLB and NLRP3-KO groups compared to the CTRL cohort (Figure 3D,F), suggesting that GLB treatment and NLRP3 deficiency had suppressed the development of colorectal tumors associated with colitis.

#### 2.2.3. Gene Expression of Inflammatory Cytokines in an AOM/DSS-Induced CRC Model

**Figure 4:**
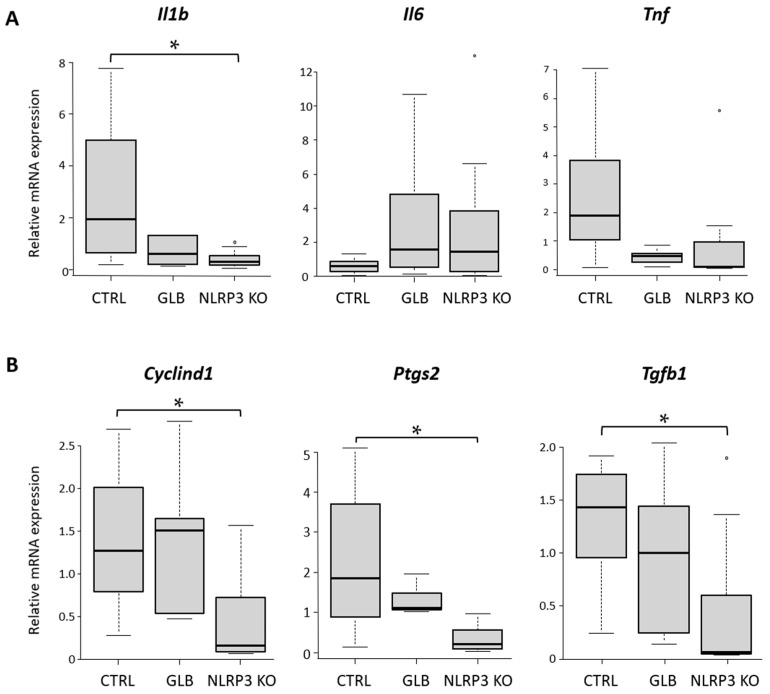
Effects of GLB and NLRP3 deficiency on gene expressions in the colon of a mouse colon tumorigenesis model. mRNA expression levels of (**A**) *Il1b*, *Il6*, and *Tnf*, and of (**B**) *Cyclind1*, *Ptgs2*, and *Tgfb* in the colonic mucosa were measured by qRT-PCR with specific primers. Data represent mean ± standard error. * *p*  <  0.05. AOM, azoxymethane; CTRL, control C57BL/6J mice; DSS, dextran sodium sulfate; GLB, glyburide; KO, knockout.

The gene expression of inflammatory cytokines in the AOM/DSS-induced CRC model was investigated to assess the effects of NLRP3-KO and GLB on cytokine production. mRNA expression levels of inflammatory cytokines in colon tissue were examined in each group. In NLRP3-KO mice, *Il1b* levels were significantly down-regulated compared to those in the CTRL group (Figure 4A). However, the expression levels of *Il6* showed no significant differences among the three groups. Similarly, the levels of *Tnf* were reduced in NLRP3-KO animals, although this difference was not statistically significant. In mice treated with GLB, the levels of both *Il1b* and *Tnf* tended to be lower than those in the CTRL group, but this did not reach statistical significance. These results suggest that GLB administration and NLRP3 deficiency down-regulated both *Il1b* and *Tnf* expression levels in the colonic mucosa, thereby attenuating colitis.

Levels of *Cyclind1*, *Ptgs2* (encoding COX2), and *Tgfb1* were significantly reduced in NLRP3-KO mice compared to those in the CTRL group in the colonic mucosa (Figure 4B). *Ptgs2* expression levels were decreased in the GLB group, but this was not statistically significant. These findings suggest that treatment with GLB and lack of NLRP3 attenuated cell proliferation and carcinogenesis in the colorectum.

## 3. Discussion

The NLRP3 inflammasome serves as a critical mediator of host defenses and plays a pivotal role in maintaining intestinal homeostasis by regulating the protective functions of the intestinal epithelium and immune responses to the intestinal microbiota [9]. Evidence suggests its involvement in the onset and progression of IBD and CRC. Despite numerous studies investigating the impact of the NLRP3 inflammasome on colonic inflammation, its role in colitis remains contentious [10,11,12]. Limited research has explored its contribution to inflammation-related colon carcinogenesis. In this study, we examined the effects of NLRP3 deficiency or pharmacological inhibition using the reported inhibitor, GLB, in models of DSS-induced colitis and AOM/DSS-induced colon tumorigenesis. This report represents the first demonstration of the efficacy of the sulfonylurea GLB in mitigating colonic inflammation-associated tumorigenesis.

In the DSS-induced colitis model, NLRP3 deficiency resulted in severe colitis characterized by significant weight loss and colon shortening. Colonic inflammation scores, based on pathological indicators such as neutrophil infiltration and crypt disappearance, were elevated in NLRP3-KO mice, despite the down-regulation of inflammatory cytokine expression in the colonic mucosa. This finding aligns with previous research [12,13,14]. Itani et al. reported severe colitis with increased expression of Th2 cytokines in NLRP3-deficient mice in a drug-induced colitis model [14]. They found that exogenous IL-1β reduced colonic Th2 cytokine expression and ameliorated colitis, suggesting that decreased IL-1β levels altered the immune status and exacerbated colitis.

The administration of GLB in our study also led to a tendency for weight loss and colon shortening in the experimental mice, but there was no significant difference in colonic inflammation scores between the GLB and CTRL groups. GLB has been recognized as an NLRP3 inhibitor and is reported to possess the ability to decrease the production of inflammatory cytokines [8,15]. The difference between NLRP3 deficiency and GLB administration appeared to be due to the inhibitory effect on IL-1β. Another reason might be that GLB affects other pathways besides NLRP3. Recent studies have reported the protective effects of GLB in dinitrobenzene sulfonic acid-induced colitis by multiple actions, including blocking cystic fibrosis transmembrane conductance regulator channels on mast cells, scavenging free radicals, and anti-inflammatory properties [16].

Interestingly, in the AOM/DSS-induced CRC model, NLRP3 deficiency and GLB treatment significantly reduced the number of tumors in the colorectum. Although *Il6* was not markedly different from the control, the expression of inflammatory cytokines such as *Il1b* and *Tnf* was decreased in the colonic mucosa. In contrast to the present study, previous reports have indicated that NLRP3 deficiency leads to colorectal tumor progression [12,17]. This inconsistency might be caused by differences in the experimental protocols, including the administration period and concentration of agents involved.

In our study, we investigated the impact of NLRP3 deficiency and inhibition on colorectal tumorigenesis, with a particular focus on the reduced levels of *Ptgs2* in the colonic mucosa. COX2, encoded by the *Ptgs2* gene, is implicated in Wnt signaling activation and the development of diseases such as cancer [18]. Furthermore, the administration of a COX2 inhibitor has been shown to suppress both the number and size of intestinal tumors in familial adenomatous polyposis [19]. Inflammatory cytokines, such as IL-1β and TNF-α, have the capacity to induce COX2 expression and prostaglandin E2 production in colon cancer-associated fibroblasts. The activation of stromal COX2 signaling, in turn, promotes colon cancer proliferation and invasion [20]. Cyclin D1 and TGF-β1 are also known to play roles in CRC development and progression [21,22]. Cyclin D1, a key cell cycle regulator, is often overexpressed in human CRC through COX2 signal activation [21]. Therefore, the down-regulation of inflammatory cytokines due to NLRP3 deficiency or inhibition may result in the inhibition of COX2 signal activation, subsequently leading to the suppression of colorectal tumor development.

GLB is a medication for diabetes mellitus and the control of blood glucose level might have effects on the development of colorectal tumors because insulin resistance is known to increase the risk of CRC [23]. However, the mice used in this study were not by nature diabetic. We examined and compared blood glucose levels in AOM/DSS-administered mice, revealing that there was no significant difference among the experimental groups. Other anti-tumor activities of GLB have been reported previously. The proliferation of endometrial adenocarcinoma and breast cancer cells was reduced by GLB treatment through blocking the potassium channels [24,25]. GLB also activated the reactive oxygen species-dependent c-Jun N-terminal kinase pathway, which was thought to be one of the important mechanisms providing protection against malignancy [26,27]. Colorectal tumorigenesis in this study might also have been suppressed through the anti-cancer effects of GLB.

Recently, various inhibitors of the NLRP3 inflammasome have been identified and developed, including GLB, MCC950, OLT177, and selnoflast [28]. MCC950 is the most used inhibitor and has been reported to ameliorate spontaneous and DSS-induced colitis [4,29]. MCC950 was also tested as a treatment for rheumatoid arthritis in a phase II clinical trial, but this failed due to hepatotoxicity [30]. GLB is the first drug shown to inhibit NLRP3 inflammasome activity and acts upstream of cryopyrin and downstream of P2X7 receptors to suppress NLRP3 inflammasome activation [8]. GLB has been reported to alleviate the symptoms of several diseases, such as acute pancreatitis and cutaneous leishmaniasis, in an NLRP3-dependent manner [31,32]. In this study, for the first time, GLB, a drug used in the treatment of type 2 diabetes in clinical practice worldwide, was found to have the ability to suppress the development of CRC.

## 4. Materials and Methods

### 4.1. Chemicals, Mice, and Diet

AOM was purchased from Wako Pure Chemical Co. (Osaka, Japan). DSS was purchased from MP Biomedicals LLC (Aurora, OH, USA). Male C57BL/6J mice and NLRP3-KO mice with a C57BL/6J background, B6.129S6-*Nlrp3^tm1Bhk^*/J (Strain 021302), aged 10 weeks, were obtained from Jackson Laboratory (Bar Harbor, ME, USA). All animals were housed in plastic cages under controlled conditions of humidity (50% ± 10%), light (12/12 h light/dark cycle), and temperature (23 °C ± 2 °C) with free access to water and food. CRF-1 (Oriental Yeast Co., Ltd., Tokyo, Japan) and CRF-1 mixed with GLB (a free form, 8 mg/kg, Oriental Yeast Co., Ltd.) were used for the diet. All procedures were conducted in accordance with the National Institutes of Health Guide for the Care and Use of Laboratory Animals and with the Guidelines for the Care and Use of Animals established by the Animal Care and Use Committee of Gifu University (Gifu, Japan). The experimental protocol was approved by the Committee of Institutional Animal Experiments of Gifu University (approval code AG-P-C-20230011).

### 4.2. Experimental Procedures

The mice were divided into three groups: a control C57BL/6J (CTRL) cohort, NLRP3-KO mice fed a control diet, and a GLB group administered a diet containing 8 mg/kg GLB. In the DSS-induced colitis model (Figure 1A), 2% DSS was administered in the drinking water for 7 days. Then, on the 8th day, all the mice were sacrificed, and their colons were dissected. In the AOM/DSS-induced CRC model, 10 mg/kg AOM was intraperitoneally injected on the first day of the experiment. One week after AOM injection, 2% DSS was administered in the drinking water for 7 days, followed by switching to normal water. The mice were sacrificed at 21 weeks from the start of the experiment, and their colons were dissected. The colons were washed with PBS and measured from the ileocecal junction to the anal verge. They were sectioned longitudinally, and colorectal tumors were visually counted. The organs, including the colon, were fixed in 10% buffered formalin for histopathological studies.

### 4.3. Histopathological Analysis

Following fixation in formalin for at least 24 h, the colons were rolled into ‘Swiss rolls’ and embedded in paraffin. A histopathological analysis was conducted on paraffin-embedded sections after hematoxylin and eosin (H&E) staining. Inflammation scores were established for the colonic mucosa according to a prior study [33].

### 4.4. RNA Extraction and Quantitative Real-Time PCR

The proximal colonic mucosa of the experimental mice was scraped, and RNA extraction was carried out using the PureLink RNA Mini Kit (Invitrogen, Carlsbad, CA, USA) following the manufacturer’s instructions. Complementary DNA (cDNA) was synthesized from each RNA sample using the High-Capacity cDNA Reverse Transcription Kit (Applied Biosystems, Foster City, CA, USA). Primers for *Cyclind1*, *Il1b*, *Il6*, *Ptgs2*, *Tgfb1*, *Tnf*, and *18s* were employed for amplification. Primer sequences used to amplify specific genes have been previously reported [34,35,36]. Real-time PCR was performed using the LightCycler (Roche Diagnostics Co., Indianapolis, IN, USA) with FastStart Essential DNA Green Master (Roche Diagnostics Co.). Gene expression levels were normalized to those of 18s.

### 4.5. Statistical Analysis

Differences between the groups were assessed using the Kruskal–Wallis test, and Steel’s tests were conducted between the groups to determine statistical significance. Tumor incidence was analyzed using Fisher’s exact test. A *p*-value of < 0.05 was considered statistically significant. Data were analyzed using R software, version 4.3.2 (The R Foundation for Statistical Computing, Vienna, Austria).

## 5. Conclusions

In conclusion, the findings of this study support the possibility that inhibition or KO of NLRP3 suppresses colorectal tumor development caused by chronic inflammation, although deletion of NLRP3 may exacerbate acute colitis. Since GLB has been used in clinical practice for a prolonged period, clinical studies can be conducted to investigate the usefulness of this drug for CRC chemoprevention in patients with IBD. Moreover, further research should be conducted to clarify how GLB suppresses colorectal carcinogenesis through means other than NLRP3 inhibition, which may lead to the elucidation of important mechanisms of inflammation-related colorectal cancer development and progression.

## Data Availability

All data that support the findings of this study are available within the article.

## References

[1] Sung H., Ferlay J., Siegel R.L., Laversanne M., Soerjomataram I., Jemal A., Bray F. (2021). Global Cancer Statistics 2020: GLOBOCAN Estimates of Incidence and Mortality Worldwide for 36 Cancers in 185 Countries. CA Cancer J. Clin..

[2] Ng S.C., Shi H.Y., Hamidi N., Underwood F.E., Tang W., Benchimol E.I., Panaccione R., Ghosh S., Wu J.C.Y., Chan F.K.L. (2017). Worldwide Incidence and Prevalence of Inflammatory Bowel Disease in the 21st Century: A Systematic Review of Population-Based Studies. Lancet.

[3] Schroder K., Tschopp J. (2010). The Inflammasomes. Cell.

[4] Wang S.-L., Zhang M.-M., Zhou H., Su G.-Q., Ding Y., Xu G.-H., Wang X., Li C.-F., Huang W.-F., Yi L.-T. (2023). Inhibition of NLRP3 Attenuates Sodium Dextran Sulfate-Induced Inflammatory Bowel Disease through Gut Microbiota Regulation. Biomed. J..

[5] Shi F., Wei B., Lan T., Xiao Y., Quan X., Chen J., Zhao C., Gao J. (2021). Low NLRP3 Expression Predicts a Better Prognosis of Colorectal Cancer. Biosci. Rep..

[6] Wang H., Wang Y., Du Q., Lu P., Fan H., Lu J., Hu R. (2016). Inflammasome-Independent NLRP3 Is Required for Epithelial-Mesenchymal Transition in Colon Cancer Cells. Exp. Cell Res..

[7] Luzzatto L., Seneca E. (2014). G6PD Deficiency: A Classic Example of Pharmacogenetics with on-Going Clinical Implications. Br. J. Haematol..

[8] Lamkanfi M., Mueller J.L., Vitari A.C., Misaghi S., Fedorova A., Deshayes K., Lee W.P., Hoffman H.M., Dixit V.M. (2009). Glyburide Inhibits the Cryopyrin/Nalp3 Inflammasome. J. Cell Biol..

[9] Shao B.-Z., Wang S.-L., Pan P., Yao J., Wu K., Li Z.-S., Bai Y., Linghu E.-Q. (2019). Targeting NLRP3 Inflammasome in Inflammatory Bowel Disease: Putting out the Fire of Inflammation. Inflammation.

[10] Zeng B., Huang Y., Chen S., Xu R., Xu L., Qiu J., Shi F., Liu S., Zha Q., Ouyang D. (2022). Dextran Sodium Sulfate Potentiates NLRP3 Inflammasome Activation by Modulating the KCa3.1 Potassium Channel in a Mouse Model of Colitis. Cell Mol. Immunol..

[11] Wagatsuma K., Nakase H. (2020). Contradictory Effects of NLRP3 Inflammasome Regulatory Mechanisms in Colitis. Int. J. Mol. Sci..

[12] Zaki M.H., Boyd K.L., Vogel P., Kastan M.B., Lamkanfi M., Kanneganti T.-D. (2010). The NLRP3 Inflammasome Protects against Loss of Epithelial Integrity and Mortality during Experimental Colitis. Immunity.

[13] Hirota S.A., Ng J., Lueng A., Khajah M., Parhar K., Li Y., Lam V., Potentier M.S., Ng K., Bawa M. (2011). NLRP3 Inflammasome Plays a Key Role in the Regulation of Intestinal Homeostasis. Inflamm. Bowel Dis..

[14] Itani S., Watanabe T., Nadatani Y., Sugimura N., Shimada S., Takeda S., Otani K., Hosomi S., Nagami Y., Tanaka F. (2016). NLRP3 Inflammasome Has a Protective Effect against Oxazolone-Induced Colitis: A Possible Role in Ulcerative Colitis. Sci. Rep..

[15] Hill J.R., Coll R.C., Sue N., Reid J.C., Dou J., Holley C.L., Pelingon R., Dickinson J.B., Biden T.J., Schroder K. (2017). Sulfonylureas as Concomitant Insulin Secretagogues and NLRP3 Inflammasome Inhibitors. ChemMedChem.

[16] Chidrawar V., Alsuwayt B. (2021). Defining the Role of CFTR Channel Blocker and ClC-2 Activator in DNBS Induced Gastrointestinal Inflammation. Saudi Pharm. J..

[17] Allen I.C., TeKippe E.M., Woodford R.-M.T., Uronis J.M., Holl E.K., Rogers A.B., Herfarth H.H., Jobin C., Ting J.P.-Y. (2010). The NLRP3 Inflammasome Functions as a Negative Regulator of Tumorigenesis during Colitis-Associated Cancer. J. Exp. Med..

[18] Telliez A., Furman C., Pommery N., Hénichart J.-P. (2006). Mechanisms Leading to COX-2 Expression and COX-2 Induced Tumorigenesis: Topical Therapeutic Strategies Targeting COX-2 Expression and Activity. Anticancer. Agents Med. Chem..

[19] Sinicrope F.A., Half E., Morris J.S., Lynch P.M., Morrow J.D., Levin B., Hawk E.T., Cohen D.S., Ayers G.D., Stephens L.C. (2004). Cell Proliferation and Apoptotic Indices Predict Adenoma Regression in a Placebo-Controlled Trial of Celecoxib in Familial Adenomatous Polyposis Patients. Cancer Epidemiol. Biomark. Prev..

[20] Zhu M., Zhu Y., Lance P. (2013). TNFα-Activated Stromal COX-2 Signalling Promotes Proliferative and Invasive Potential of Colon Cancer Epithelial Cells. Cell Prolif..

[21] Arber N., Hibshoosh H., Moss S.F., Sutter T., Zhang Y., Begg M., Wang S., Weinstein I.B., Holt P.R. (1996). Increased Expression of Cyclin D1 Is an Early Event in Multistage Colorectal Carcinogenesis. Gastroenterology.

[22] Singh S., Gouri V., Samant M. (2023). TGF-β in Correlation with Tumor Progression, Immunosuppression and Targeted Therapy in Colorectal Cancer. Med. Oncol..

[23] Shirakami Y., Shimizu M., Kubota M., Araki H., Tanaka T., Moriwaki H., Seishima M. (2014). Chemoprevention of Colorectal Cancer by Targeting Obesity-Related Metabolic Abnormalities. World J. Gastroenterol..

[24] Núñez M., Medina V., Cricco G., Croci M., Cocca C., Rivera E., Bergoc R., Martín G. (2013). Glibenclamide Inhibits Cell Growth by Inducing G0/G1 Arrest in the Human Breast Cancer Cell Line MDA-MB-231. BMC Pharmacol. Toxicol..

[25] Erdem Kış E., Tiftik R.N., Al Hennawi K., Ün İ. (2022). The Role of Potassium Channels in the Proliferation and Migration of Endometrial Adenocarcinoma HEC1-A Cells. Mol. Biol. Rep..

[26] Qian X., Li J., Ding J., Wang Z., Duan L., Hu G. (2008). Glibenclamide Exerts an Antitumor Activity through Reactive Oxygen Species-c-Jun NH2-Terminal Kinase Pathway in Human Gastric Cancer Cell Line MGC-803. Biochem. Pharmacol..

[27] Yan B., Peng Z., Xing X., Du C. (2017). Glibenclamide Induces Apoptosis by Activating Reactive Oxygen Species Dependent JNK Pathway in Hepatocellular Carcinoma Cells. Biosci. Rep..

[28] Chen Q.-L., Yin H.-R., He Q.-Y., Wang Y. (2021). Targeting the NLRP3 Inflammasome as New Therapeutic Avenue for Inflammatory Bowel Disease. Biomed. Pharmacother..

[29] Perera A.P., Fernando R., Shinde T., Gundamaraju R., Southam B., Sohal S.S., Robertson A.A.B., Schroder K., Kunde D., Eri R. (2018). MCC950, a Specific Small Molecule Inhibitor of NLRP3 Inflammasome Attenuates Colonic Inflammation in Spontaneous Colitis Mice. Sci. Rep..

[30] Mangan M.S.J., Olhava E.J., Roush W.R., Seidel H.M., Glick G.D., Latz E. (2018). Targeting the NLRP3 Inflammasome in Inflammatory Diseases. Nat. Rev. Drug Discov..

[31] Carvalho A.M., Novais F.O., Paixão C.S., de Oliveira C.I., Machado P.R.L., Carvalho L.P., Scott P., Carvalho E.M. (2020). Glyburide, a NLRP3 Inhibitor, Decreases Inflammatory Response and Is a Candidate to Reduce Pathology in Leishmania Braziliensis Infection. J. Investig. Dermatol..

[32] York J.M., Castellanos K.J., Cabay R.J., Fantuzzi G. (2014). Inhibition of the Nucleotide-Binding Domain, Leucine-Rich Containing Family, Pyrin-Domain Containing 3 Inflammasome Reduces the Severity of Experimentally Induced Acute Pancreatitis in Obese Mice. Transl. Res..

[33] Wirtz S., Neufert C., Weigmann B., Neurath M.F. (2007). Chemically Induced Mouse Models of Intestinal Inflammation. Nat. Protoc..

[34] Kato J., Shirakami Y., Mizutani T., Kubota M., Sakai H., Ibuka T., Shimizu M. (2020). Alpha-Glucosidase Inhibitor Voglibose Suppresses Azoxymethane-Induced Colonic Preneoplastic Lesions in Diabetic and Obese Mice. Int. J. Mol. Sci..

[35] Shirakami Y., Kato J., Maeda T., Ideta T., Imai K., Sakai H., Shiraki M., Shimizu M. (2023). Skeletal Muscle Atrophy Is Exacerbated by Steatotic and Fibrotic Liver-Derived TNF-α in Senescence-Accelerated Mice. J. Gastroenterol. Hepatol..

[36] Miyazaki T., Shirakami Y., Mizutani T., Maruta A., Ideta T., Kubota M., Sakai H., Ibuka T., Genovese S., Fiorito S. (2021). Novel FXR Agonist Nelumal A Suppresses Colitis and Inflammation-Related Colorectal Carcinogenesis. Sci. Rep..

